# H_2_S-mediated balance regulation of stomatal and non-stomatal factors responding to drought stress in Chinese cabbage

**DOI:** 10.1093/hr/uhac284

**Published:** 2022-12-23

**Authors:** Wenze Zhang, Lei Wang, Liping Zhang, Xiangqun Kong, Jiao Zhang, Xin Wang, Yanxi Pei, Zhuping Jin

**Affiliations:** School of Life Science and Shanxi Key Laboratory for Research and Development of Regional Plants, Shanxi University, Taiyuan, Shanxi Province 030032, China; School of Life Science and Shanxi Key Laboratory for Research and Development of Regional Plants, Shanxi University, Taiyuan, Shanxi Province 030032, China; Taiyuan Yuying High School, Taiyuan, Shanxi Province 030009, China; School of Life Science and Shanxi Key Laboratory for Research and Development of Regional Plants, Shanxi University, Taiyuan, Shanxi Province 030032, China; School of Life Science and Shanxi Key Laboratory for Research and Development of Regional Plants, Shanxi University, Taiyuan, Shanxi Province 030032, China; School of Life Science and Shanxi Key Laboratory for Research and Development of Regional Plants, Shanxi University, Taiyuan, Shanxi Province 030032, China; School of Life Science and Shanxi Key Laboratory for Research and Development of Regional Plants, Shanxi University, Taiyuan, Shanxi Province 030032, China; School of Life Science and Shanxi Key Laboratory for Research and Development of Regional Plants, Shanxi University, Taiyuan, Shanxi Province 030032, China; School of Life Science and Shanxi Key Laboratory for Research and Development of Regional Plants, Shanxi University, Taiyuan, Shanxi Province 030032, China

## Abstract

Increased evidence has shown that hydrogen sulfide (H_2_S), a novel gasotransmitter, could enhance drought resistance in plants by inducing stomatal closure, with concurrent enhancement of photosynthetic efficiency, but little is known about the mechanism behind this contradictory phenomenon. This study examined the regulating mechanism of H_2_S in response to drought stress from stomatal and non-stomatal factors in Chinese cabbage. The results showed that exogenous H_2_S could increase the accumulation of photosynthetic pigments and alleviate the damage caused by drought stress. It also regulated the expression in transcriptional level and the activity of ribulose 1,5-bisphosphate carboxylase/oxygenase (BrRuBisCO) under drought stress. The large subunit of BrRuBisCO was found to be modified by S-sulfhydration, which might be the reason for its increased enzyme activity. The fluxes of Cl^−^, K^+^, and H^+^ in the guard cells were detected by non-invasive micro-test techniques while under drought stress. The results indicated that H_2_S signaling induced a transmembrane Cl^−^ and H^+^ efflux and inhibited K^+^ influx, and the Cl^−^ channel was the main responders for H_2_S-regulated stomatal movement. In conclusion, H_2_S signal not only activated the ion channel proteins located in the guard cell membrane to induce stomatal closure, but also regulated the transcriptional expression and the activity of RuBisCO, a non-stomatal factor to enhance the photosynthetic efficiency of leaves. There is therefore a beneficial balance between the regulation of H_2_S signaling on stomatal factors and non-stomatal factors due to drought stress, which needs to be better understood to apply it practically to increase crop yields.

## Introduction

Drought stress has become a major limiting factor for crop production worldwide, exceeding the sum of all other cropping adversity factors in China [1]. Photosynthesis is essential for plant growth and development, and drought stress can reduce crop yields by inhibiting photosynthesis. It is reported that the inhibitory effect of drought on photosynthesis was divided into stomatal-limited and non-stomatal-limited factors [2]. Under mild drought, the stomata of leaves tend to be closed to reduce water loss, resulting in inefficient gas exchange and reduced photosynthetic rate, showing stomatal limitation of photosynthesis. Under persistent or severe drought, the structure of chloroplasts and stromal sheets is destroyed, the efficiency of photosynthetic electron transfer is reduced, and the activity of ribulose 1,5-bisphosphate carboxylase/oxygenase (RuBisCO) is inhibited, eventually leading to photosynthesis rate drop or non-stomatal limitation of photosynthesis [3].

Chinese cabbage (*Brassica rapa* L. ssp. *pekinensis*), a biennial herb of the Brassica family, is a popular leafy vegetable crop cultivated worldwide. The rosette stage of Chinese cabbage growth is the most vigorous stage of vegetative growth, requiring sufficient water and fertilizer supply, so drought conditions will seriously limit normal development and growth, damaging its yield and quality [4, 5]. It is therefore important to understand the key factors that limit the photosynthetic efficiency of Chinese cabbage under drought-induced stress and to take effective measures to resist adverse stress and maintain its yield and quality.

Hydrogen sulfide (H_2_S) was recognized as the third gasotransmitter after nitric oxide and carbon monoxide in mammals and plants [6]. Recent studies have shown that sulfhydration of protein cysteine residues is an important mechanism of H_2_S signal transduction in plants, where the cysteine thiol (-RSH) is sulfhydrated into a persulfide thiol (-RSSH) [7, 8]. This modification may modulate protein activity and function and is measured by a biotin switch assay (BSA) [9]. The important physiological functions of H_2_S as a signaling molecule in higher plants have been gradually identified as promoting seed germination, root growth, delaying flower organ opening and senescence, activating the antioxidant enzyme system, and enhancing the resistance of the plant to various stresses [10–14]. The stomatal closure function induced by H_2_S was revealed firstly in thale cress (*Arabidopsis thaliana)*, broad beans (*Vicia faba*), and impatiens (*Impatiens walleriana*) [15, 16]. Follow-up studies have improved the understanding of the regulation of stomatal movement by H_2_S, which was involved in the pathway of stomatal closure induced by endogenously phytohormones, such as abscisic acid (ABA), ethylene salicylic acid, and jasmonic acid [14, 17–19]. At the same time, H_2_S may affect the movement of microfilaments and microtubules through secondary signals, indirectly inducing the movement of guard cells and promoting stomatal closure [20]. It may also trigger sulfhydration to regulate stomatal movement. The Snf1-related protein kinase 2.6 (SnRK2.6) and ABI4 sulfhydration are critical in response to ABA during stomatal closure [21, 22].

Recently, studies have shown that H_2_S achieves stomatal closure by affecting the activity of K^+^ and Cl^−^ channels in guard cells [23, 24]. Using the non-invasive micro-test technique (NMT) bioassays that measure real-time flux changes of specific molecules and ions in individual cells *in vivo*, K^+^ channels are the main responder of stomatal movement to H_2_S, which provided a more comprehensive understanding of the mechanism of H_2_S-induced stomatal closure [25]. NMT is a technology to detect physiological functions *in vivo*. Just as DNA sequencing technology reveals genetic information by detecting deoxyribonucleic acid, NMT reveals the physiological function *in vivo* by detecting the concentration of ionic molecules and their gradient. Ionic and molecular homeostasis is one of the common features of all life, and it is a dynamic equilibrium. This homeostasis is achieved by maintaining ion and molecular concentration gradients on both sides of each biofilm. NMT reveals the homeostasis of ionic molecules in living materials and its related physiological function mechanism by detecting the concentration gradient formed by ionic molecules moving across the membrane. This technique can non-invasively measure various ions and small molecules *in situ* with high levels of temporal and spatial resolution.

The main carboxylase in photosynthesis RuBisCO is the most abundant enzyme in the biosphere and one of the best-characterized [26]. The RuBisCO from algae and higher plants consists of eight large subunits (LSU) and eight small subunits (SSU) assembled with the assistance of molecular chaperones into the full enzyme structure of L8S8. There is an active site on the large subunit and a possible regulatory role for the small subunit [27]. Previous studies on this enzyme have been difficult due to its large molecular weight and complex folding and assembly pathways. The researchers co-expressed seven chloroplast molecular chaperones that helped RuBisCO fold and assemble in *Escherichia coli (E. coli)*, resulting in functional RuBisCO.

Physiological concentrations of H_2_S can enhance photosynthetic efficiency by increasing the activity of the key photosynthetic enzyme RuBisCO, which can accelerate plant growth and ultimately increase yield [28–30]. However, the in-depth mechanism of H_2_S promoting RuBisCO activity is still unclear as it participates in the opposing processes of inducing stomatal closure and enhancing photosynthesis at the same time. There is a need to understand how stomatal closure is induced under drought stress to reduce damage to plants, while promoting non-stomatal limiting factors to achieve higher photosynthetic efficiency. This study combined ion homeostasis and protein thiolation modification to explore how H_2_S regulated the balance of stomatal and non-stomatal factors in Chinese cabbage under drought stress.

## Results

### H_2_S increased the accumulation of photosynthetic pigments to facilitate growth under drought stress

**Figure 1:**
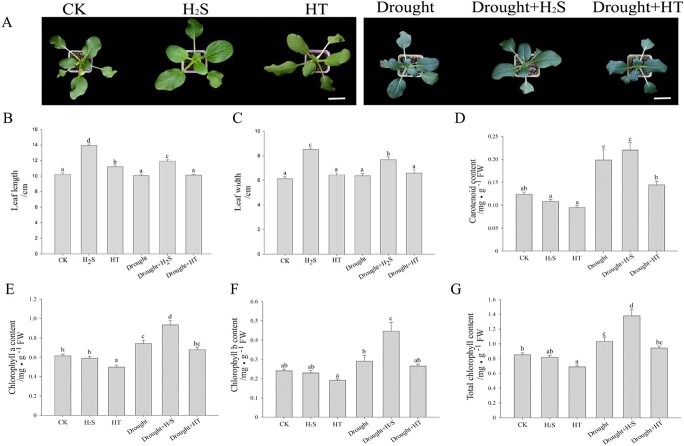
Effect of H_2_S on growth and photosynthetic pigments. (**A**) Phenotype of one-month-old cabbage fumigated with H_2_S or HT under normal or drought conditions; to determine (**B**) leaf length; (**C**) leaf width; (**D**) carotenoid content; (**E**) chlorophyll a content; (**F**) chlorophyll b content; and (**G**) total chlorophyll content under the different treatments. Error bars indicate the standard error of three biological replicates and different lowercase letters are significantly different among treatments (*P* < 0.05).

In Fig. 1A–C, results showed that the leaf length and leaf width of H_2_S and Drought+H_2_S treated groups were significantly higher than those of the CK and Drought groups. Various photosynthetic pigments were detected, which are essential indicators in response to drought stress, but, the plants in different treatment groups showed similar patterns. The content of chlorophyll a (Chl a) was the highest and carotenoids (Caro) was the lowest, with the ratio of Chl a to chlorophyll b (Chl b) content close to 7:3, as shown in Fig. 1D–F. The total chlorophyll content decreased significantly in the HT group compared to the CK group and increased dramatically in the Drought+H_2_S group compared to the Drought group, which indicated that H_2_S could increase the total content of chlorophyll as in Fig. 1G. Exogenous physiological concentration of H_2_S could increase the accumulation of photosynthetic pigments in cabbage to alleviate drought stress.

### H_2_S enhanced the photosynthetic rate and water use efficiency (*WUE*) under drought stress

**Figure 2:**
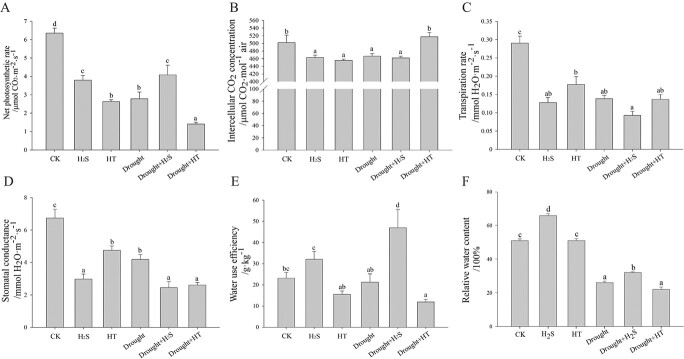
H_2_S increased the photosynthetic rate and *WUE* in response to drought stress. The (**A**) net photosynthetic rate (*Pn*), (**B**) intercellular CO_2_ concentration (*Ci*), (**C**) transpiration rate (*Tr*), (**D**) stomatal conductance (*Gs*), (**E**) water utilization efficiency (*WUE*), and (**F**) relative water content (*RWC*) were determined. Error bars indicate the standard error of three biological replicates and different lowercase letters are significantly different among treatments (*P* < 0.05).

To further understand the effect of H_2_S on the photosynthetic system, the photosynthetic indicators of cabbage were analysed under different treatments. Under normal conditions, as shown in Fig. 2, net photosynthetic rate (*Pn*), intercellular CO_2_ concentration (*Ci*), transpiration rate (*Tr*), and stomatal conductance (*Gs*) were all significantly reduced after H_2_S treatment. In contrast, removing endogenous H_2_S with HT treatment resulted in a significant decrease in *Pn* in Fig. 2A and a significant increase in *Gs* in Fig. 2D, which implied that H_2_S might regulate photosynthesis through non-stomatal factors. Under drought stress, *Pn* increased significantly, and *Ci* values remained unchanged as shown in Fig. 2B, suggesting that the promotion of plant photosynthesis by H_2_S under drought stress may be related to non-stomatal factors. The *Ci* values of the Drought+HT group were significantly higher than those of the Drought group, but the *Pn* values were lower, suggesting that H_2_S deficiency may limit some non-stomatal factors and inhibit photosynthesis. In contrast, all the *Ci*, *Tr*, and *Gs* reduced significantly, and *Pn* decreased extremely significantly, which indicated a decrease in the *Pn* of plants under drought stress was limited by stomatal factors. The Drought+H_2_S group showed a significant decline in *Gs* and further closure of stomata compared with the Drought group. To verify the effect of H_2_S on stomatal movement, *WUE*, which is theoretically the ratio of *Pn* to *Tr*, was recorded and indicated an extremely significant enhancement by H_2_S under drought stress, as shown in Fig. 2E, which was consistent with the calculated data. Fig. 2F shows that the relative water content (*RWC*) of cabbage leaves was significantly elevated by H_2_S either in normal conditions or under drought stress. It is suggested that H_2_S may retain water content by regulating stomatal movement and enhancing the resistance to drought stress through stomatal and non-stomatal factors at the same time.

### H_2_S upregulated the expression of BrRuBisCO in transcriptional level under drought stress

**Figure 3:**
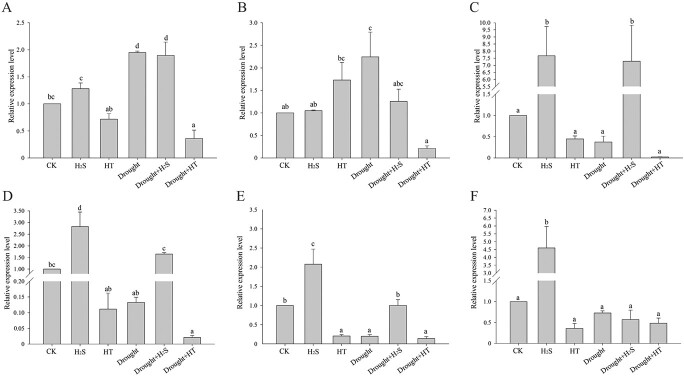
Effect of H_2_S on the large and small subunits of BrRuBisCO at the transcriptional level. (**A**) Expression of the large subunit BrRBCL and the small subunits (**B**) BrRBCS74, (**C**) BrRBCS24, (**D**) BrRBCS06, (**E**) BrRBCS31/F1, and (**F**) BrRBCS27/29 of BrRuBisCO were detected under different treatment conditions. Error bars indicate the standard error of three biological replicates and different lowercase letters are significantly different among treatments (*P* < 0.05).

As a key enzyme in photosynthesis, BrRuBisCO was chosen to demonstrate the effect of the H_2_S signal on the non-stomatal factors. The changes in the expression of genes encoded by the large subunit BrRBCL and small subunit BrRBCS of BrRuBisCO were determined under different treatments. Fig. 3 showed that the expression of all genes, except *BrRBCL* and *BrRBCS74*, was significantly increased after H_2_S fumigation under normal conditions. The expression of *BrRBCS24*, *BrRBCS06*, and *BrRBCS31/F1* was elevated in the H_2_S-treated group under drought stress, while *BrRBCL*, *BrRBCS74*, and *BrRBCS27/29* showed no meaningful change, but declined significantly after the removal of endogenous H_2_S using HT suggesting that H_2_S upregulated the transcriptional levels of BrRuBisCO in response to drought stress.

### H_2_S sulfhydrated BrRBCL and elevated the activity of RuBisCO

**Figure 4:**
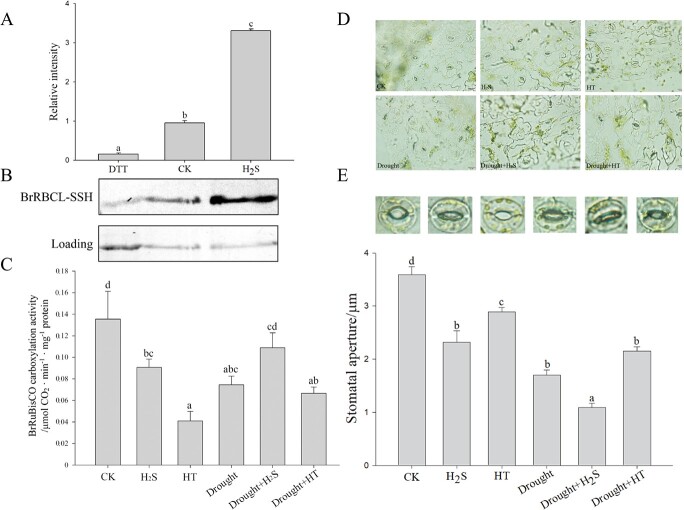
Effect of H_2_S on RuBisCO enzyme at the post-translational level and stomata of cabbage leaves under different treatments. (**A**) Grayscale analysis of western blot bands. (**B**) The effect of H_2_S on S-sulfhydration of pET-28a-BrRBCL protein from *E. coli* BL21 (DE3). H_2_S: BrRBCL treated with 2 mmol·L^−1^ NaHS; DTT: BrRBCL treated with 2 mmol·L^−1^ DTT. (**C**) Determination of RuBisCO carboxylation activity in Chinese cabbage seedlings under different treatments. (**D**) Changes in stomatal aperture of each group were observed under an optical microscope. Bars = 20 μm. (**E**) Average stomatal opening of leaves under different treatments. Error bars indicate the standard error of three biological replicates and different lowercase letters are significantly different among treatments (*P* < 0.05).

Including the active site of RuBisCO holoenzyme, BrRBCL fusion protein was expressed and purified by the prokaryotic system with His-tag from *E. coli* BL21(DE3) and then the S-sulfhydration modification was detected using the biotin-switch method. Fig. 4 shows that BrRBCL was S-sulfhydrated after treatment with H_2_S. The western blotting signal clearly showed a significant increase in S-sulfhydration after exogenous H_2_S treatment and an obvious decrease with dithiothreitol (DTT) treatment, as seen in Fig. 4A and B. For further validation *in vivo*, the RuBisCO carboxylation activity of the total proteins in the different treatment groups was measured. Fig. 4C showed that under normal conditions, the carboxylation activities of RuBisCO in both HT and H_2_S groups were significantly decreased compared to CK, indicating that the absence of endogenous H_2_S significantly inhibited the enzyme carboxylation activity in normal growth conditions. Conversely, the carboxylation activities of RuBisCO were promoted significantly with H_2_S under drought stress, so the H_2_S signal might regulate the activity of RuBisCO in diverse ways under different conditions.

### H_2_S induced stomatal closure in cabbage leaves

To determine the effect of H_2_S on stomatal movement in Chinese cabbage, the stomatal apertures under normal and drought conditions were measured and compared (Fig. 4D). Under normal conditions, the stomatal aperture after H_2_S treatment was significantly lower than that of CK group. All stomatal apertures were reduced under drought stress compared to normal conditions. There was an obvious stomatal movement not only with exogenous supplementation but also with endogenous clearance of H_2_S (Fig. 4E) under drought stress. These results were consistent with that of stomatal conductance measurements (Fig. 2D), suggesting H_2_S induced stomatal closure in cabbage leaves in response to drought stress.

### H_2_S triggered ion flows through guard cell membrane in response to drought stress

The stomatal factors of H_2_S on photosynthesis in Chinese cabbage were analysed using NMT to determine the ion flows in the guard cell membrane. The flow rate variation of Cl^−^, K^+^, and H^+^ was analysed in real time for six minutes and the mean flow rate was calculated under different conditions.

**Figure 5:**
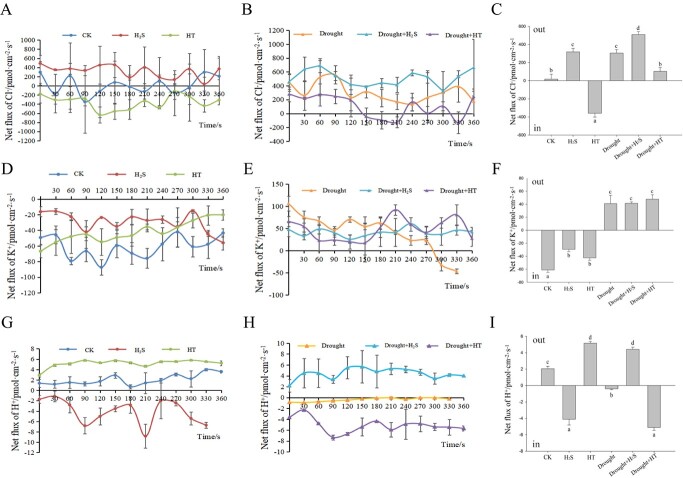
Effect of H_2_S on the net flow of Cl^−^, K^+^, and H^+^ in guard cell membranes under drought stress. (**A**–**C**) Net fluxes of Cl^−^, (**D**–**F**) K^+^, and (**G**–**I**) H^+^ of guard cells in the lower epidermal tissues isolated from leaves of cabbage under drought conditions. Error bars indicate the standard error of three biological replicates and different lowercase letters are significantly different among treatments (*P* < 0.05).

As shown in Fig. 5A–C, under normal conditions, the net Cl^−^ flow rate in the guard cell membrane fluctuated steadily during the recording period and the mean value showed an outward flow at about 17 pmol·cm^−2^·s^−1^ and H_2_S treatment significantly enhanced Cl^−^ efflux with a flow rate value of 318 pmol·cm^−2^·s^−1^. Accordingly, there was a significant inward flow of Cl^−^ following the removal of endogenous H_2_S with HT. Under drought stress, the same Cl^−^ flux trends were shown, but all treatment groups had significant increases compared to the corresponding treatment groups under normal conditions. The Drought group maintained a net Cl^−^ flow rate of about 305 pmol·cm^−2^·s^−1^, an increase of about 18-fold compared to the CK group. The Cl^−^ efflux was increased by 67% in the Drought+H_2_S group and decreased by 66% in the Drought+HT group based on drought stress alone, so H_2_S signal had a more significant effect on promoting Cl^−^ efflux across the defense guard cell membrane under drought stress in cabbage leaves.

As shown in Fig. 5D–F, the CK, H_2_S, and HT groups under normal conditions had consistently negative net flow values over the period evaluated and all showed continuous K^+^ inward flow. In the CK group, the mean in-flow rate of K^+^ was about 61 pmol·cm^−2^·s^−1^ and in the H_2_S group, the K^+^ in-flow was significantly reduced by about 52% compared to the CK group. The Drought, Drought+H_2_S and Drought+HT groups showed continuous K^+^ efflux, with mean flow values ranging from 40 to 48 pmol·cm^−2^·s^−1^.

Fig. 5G–I showed that under normal conditions, the net H^+^ flow rate across the guard cell membrane of cabbage leaves showed a slow outflow and fluctuated steadily, remaining at a low flow rate value of about 2 pmol·cm^−2^·s^−1^. Compared to the CK group, H_2_S treatment showed a significant increase in promoting H^+^ inward flow, with the mean value up to about 4 pmol·cm^−2^·s^−1^, while the HT group showed a significantly enhanced outflow of H^+^ with an increase of about 1.5-fold. The Drought group compared to the CK group and the Drought+HT group compared to the HT group both reflected that drought conditions caused a significant increase in H^+^ inward flow across the defense cell membrane of cabbage. Overall, the H^+^ stream showed the smallest flow rate values for each treatment condition, showing the slowest flow rate of the three ions evaluated.

In summary, from the value of the ordinate, the Cl^−^ channel may be the main target of the H_2_S signal, which can regulate the stomatal movement by adjusting the ion flow velocity and direction to change the turgor pressure.

## Discussion

The RuBisCO is an enzyme required for carbon dioxide (CO_2_) fixation in the first step of the Calvin cycle and it is also the most abundant enzyme in plants. A recent study showed that overexpression of the RuBisCO subunit leads to an increase in the amount of RuBisCO, an increase in the rate of CO_2_ assimilation and the effects of low temperature in C4 species are mitigated [31]. Sulfhydration by modifying the sulfhydryl group of cysteine residue (Cys-SH) to the hydroperoxysulfide group (Cys-SSH) and altering protein conformation and activity is a widely accepted mechanism of H_2_S signalling [7, 8]. This study focused on the analysis of BrRuBisCO from the perspective of sulfhydration modification and activity change, to obtain an effective way to improve the photosynthetic efficiency of Chinese cabbage under drought stress. The different subunits of the AtRuBisCO enzyme were prokaryotic expressed and detected the sulfhydration signal of the model plant *A. thaliana*. The results showed that the large subunit protein of AtRuBisCO termed AtRBCL in Fig. S1 (see online supplementary material) and two small subunits AtRBCS1A and AtRBCS3B in Fig. S2 (see online supplementary material) can be modified by NaHS sulfhydration. As Brassicaceae are ancient polyploid relatives of Arabidopsis [32], the sequence alignment showed that the AtRuBisCO and BrRuBisCO proteins were highly homologous, as shown in Fig. S3 (see online supplementary material). It has been shown that the large subunit is the active center of the RuBisCO holoenzyme, so BrRBCL was chosen as the representative and conducted subsequent modification detection. Fig. 4 showed a significantly increased band in S-sulfhydrated after exogenous H_2_S treatment, which can be attenuated by DTT treatment. It was verified that the carboxylation activity of RuBisCO could be significantly promoted by H_2_S *in vivo*, so H_2_S might enhance BrRuBisCO activity by the S-sulfhydration of BrRBCL, which provides a new mechanism for H_2_S-mediated BrRuBisCO resistance to drought stress.

Stomata are micro pores mainly located on leaf lower surface of terrestrial plants, surrounded by two guard cells. H_2_O and CO_2_ exchanging through stomatal pores results in stomatal movement, as the key process for drought resistance and photosynthesis. In the regulation of the closing or opening of stomata by H_2_S, guard cells respond to adverse environmental stress signals by controlling the closure or opening of stomata. Stomatal closure under drought stress is mediated by a complex signaling network involving ABA, hydrogen peroxide and nitric oxide, with ABA being significant and H_2_S being a small signaling gas molecule involved in ABA-dependent stomatal closure [25]. A substantial literature shows that H_2_S regulates the ABA signaling pathway in guard cells through the persulfation of specific targets. H_2_S acts by guarded cellular persulfation of SNF1-related and ABI4 proteins and positive regulation of the ABA signaling pathway, which is essential for controlling stomatal closure [21, 22, 33]. ABA stimulates the persulfidation of DES1 at Cys44 and Cys205 in a redox-dependent manner. Moreover, sustainable H_2_S accumulation drives persulfidation of RBOHD (NADPH oxidase, respiratory burst oxidase homolog protein D) at Cys825 and Cys890, enhancing its ability to produce reactive oxygen species [34]. The discovery of a group of proteins such as protein kinases and phosphatases involved in ABA signaling in guard cells was also reported in the persulfation proteome [35] and proved under cold stress [36].

Most researchers located the regulation of stomatal movement by H_2_S in its post-translational modifying action, but what is important is that ABA promotes stomatal closure in guard cells and inhibits stomatal opening in a process that regulates the activity of a variety of ion flows [37]. The guard cells also regulate stomatal aperture through osmotic solute uptake and loss, especially K^+^ and Cl^−^ [38]. In this study, NMT was used to determine several ion flows associated with stomatal movement in cabbage and the response of the Cl^−^ ion flow to H_2_S is much greater than that of the other ion streams. Specifically, Cl^−^ is the most sensitive to each treatment, with the largest net flow value, so it appears that Cl^−^ flow is the main osmolyte responder to H_2_S in regulating stomatal movement in response to drought stress. The Cl^−^ channel protein might function as the main target of H_2_S signal regulating the volume of guard cells in Chinese cabbage during stomatal closure. These results are consistent with a recent study, which also provided direct evidence for the activation of anion channels by H_2_S in Arabidopsis guard cells, so anion channels may be potential targets for direct action during stomatal closure [23]. For anions, Cl^−^ efflux through SLAC channels in guard cells is associated with a drought stress response dependent on ABA signaling [39, 40]. Another two recent studies have reported that K^+^ flow in Arabidopsis and tobacco may be the main targets of H_2_S action [24, 25]. It may be due to different experimental systems, varied species, and different states, the target of H_2_S may not be fixed, but the ion channel of guard cells as the target of H_2_S deserves attention and future studies of ion flow-related mutants will help to elucidate the roles between various ions during H_2_S-induced stomatal movement.

**Figure 6:**
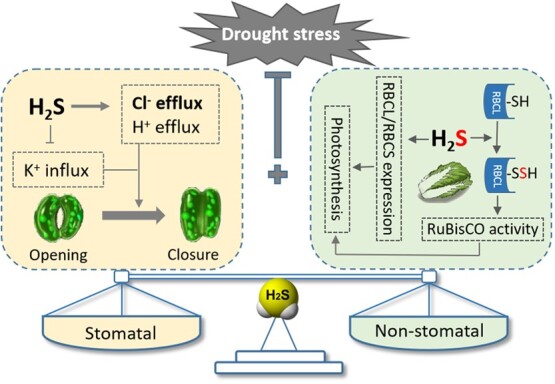
Proposed model of H_2_S balanced stomatal and non-stomatal factors in response to drought stress in Chinese cabbage. H_2_S: hydrogen sulfide; RBCL: large subunit of RuBisCO; RBCS: small subunits of RuBisCO; RuBisCO: Ribulose 1,5-bisphosphate carboxylase/oxygenase; -SH: hydrosulfuryl; Arrow end: activation; Blunt end: inactivation.

**Table 1:** The list of specific information of six treatment groups of Chinese cabbage

Group	Drought stress	NaHS (100 μmol·L^−1^)	HT(100 μmol·L^−1^)
CK	−	−	−
H_2_S	−	+	−
HT	−	−	+
Drought	+	−	−
Drought+H_2_S	+	+	−
Drought+HT	+	−	+

In fact, stomatal movement is a particularly complex process, which is regulated by a large number of internal and external factors. For example, the stomatal conductance under Drought+HT is significantly lower than that of Drought (Fig. 2D); however, the stomatal aperture of Drought+HT is the same as Drought (Fig. 4E). To more clearly show the effect of HT, we sprayed HT on leaves of *A. thaliana* and Chinese cabbage, and then detected the changes in H_2_S content in leaves of different plants. As shown in Fig. S4 (see online supplementary material), the content of H_2_S decreased sharply after applying exogenous HT, and there was significant difference between the control and HT group, respectively. Therefore, we believe that the seedling age of experimental materials in different determination systems is different (Figs 2D and 4E), which may lead to the inconsistency between experimental results and theoretical speculation strictly. Because of this, the mechanism of stomatal movement has always been a hot spot and focus in the field of stress resistance. Therefore, the challenge has remained to identify a core and unifying mechanism that can account for the regulation of stomatal aperture and conductance through H_2_S signaling mediated ion flux and carbon assimilation.

As a signaling molecule, H_2_S participates in various physiological activities through different signaling pathways. It is well known that H_2_S regulated stomatal movement response to drought stress. The ion channels of guard cell membranes are regulated and the osmotic potential and turgor pressure of guard cells are altered, leading to stomatal closure while enhancing photosynthesis. It has been puzzling that stomatal closure is a limiting factor for photosynthesis and it was unclear how H_2_S induced stomatal closure while ensuring efficient photosynthesis, but this study confirmed that under drought stress, stomata were closed by H_2_S to conserve water and the activity of RuBisCO enzyme was elevated by the S-sulfhydration of BrRBCL. The balance regulation of H_2_S between stomatal and non-stomatal factors responding to drought stress in Chinese cabbage may maximize photosynthetic efficiency as shown in Fig. 6.

**Table 2:** Different experimental treatments on the lower epidermal tissues isolated from leaves of Chinese cabbage

Group	Reagent in a petri dish
CK	3 mL MES buffer
H_2_S	3 mL MES buffer+ NaHS spatial concentration of 100 $\mu$mol·L^−1^
HT	3 mL 100 $\mu $mol·L^−1^ HT solution
Drought	3 mL 0.1 g·mL^−1^ PEG-8000 solution
Drought+H_2_S	3 mL 0.1 g·mL^−1^ PEG-8000 solution + NaHS with a spatial concentration of 100 $\mu $mol·L^−1^
Drought+HT	1.5 mL 0.2 g·mL^−1^ PEG-8000 solution +1.5 mL 200 $\mu $mol·L^−1^ HT solution

## Materials and methods

### Plant material and growth conditions

Chinese cabbage seeds (*B. rapa* L. ssp. *pekinensis*) of ‘Aiqing No.1’ were donated by Professor Jiashu Cao from the Institute of Vegetable Research at Zhejiang University, China. The seeds were sown on three layers of moistened gauze, then grown for about eight days at 23°C, relative humidity of 60%, a light intensity of 3000 lux, and a long sunshine time of 16 hours per day, with the gauze kept moist during the period. After the seeds germinated into dicotyledonous seedlings, they were colonized in matrix soil and grown under long-day 16 h/8 h conditions at 23°C.

### Plant material treatments

In this study, NaHS (sodium hydrosulfide, H_2_S donor) and HT (hypotaurine, H_2_S scavenger) were used to treat the plants, aiming to explore the physiological function of H_2_S signal in alleviating drought stress of Chinese cabbage. One-month-old cabbage seedlings were divided into the control group (CK) and the drought group (Drought) for five days. The CK group was watered normally and the Drought group was without water during this period. Then the CK group and the Drought group were divided into three groups (about 10 seedlings in each group). In the first group, 3 mL of distilled water was sprayed on seedling leaves as a control. In the second group, after spraying the leaves with 3 mL distilled water, the seedlings were placed in a closed glass cover and fumigated with 100 μmol·L^−1^ NaHS for 6 hours a day (9:00–15:00). In the third group, 3 mL of 100 μmol·L^−1^ HT solution was sprayed on seedling leaves. All treatments last for 8 days. After that, three plants were randomly selected from each treatment for observation and sampling. During the experiment, it is guaranteed that no fewer than three times of biological repetitions and technical repetitions are performed. The specific information of groups is shown in Table 1.

### Determination of leaf width and length and chlorophyll content

The leaves of plants with different treatments in Table 1 (the seedling age is about 45 days) were used for the determination of leaf width, length, and chlorophyll content. Leaf width and length were measured by the longest and widest lengths of all leaves and the average was calculated. Chlorophyll content determination method as described above [41], fresh leaves were homogenized in 95% ethanol, with attention to shading, and centrifuged at 3000 g for 10 min. The absorbance of the supernatant was measured at OD_665_, OD_649_, and OD_470_ with a UV-2100 spectrophotometer (UNIC, Texas, USA), respectively. Finally, the pigment content of each chloroplast was calculated according to the following formula. Chl a concentration: C_a_ = 13.95 × D_665−_6.88 × D_649_, Chl b concentration: C_b_ = 24.96 × D_649−_7.32 × D_665_, Caro concentration: Cx.c = (1000 × D_470_–2.05 × C_a_ − 114.8 × C_b_)/245, chlorophyll concentration: C_T_ = C_a_ + C_b_, chloroplast pigment content: [pigment concentration (mg·L^−1^) × extract volume (L)]/leaf fresh weight (g).

### Determination of relevant photosynthetic indicators and relative water contents

Various photosynthetic parameters of seedlings after different treatments in Table 1 (the seedling age is about 45 days) were detected using an SY-1020 portable photosynthesis analyser (Shijiazhuang Shiya Technology Co., Ltd., China), including *Pn*, *Ci*, *Tr*, *Gs*, and *WUE*. The measurement requires sufficient light source and stable CO_2_ content in the air without wind. The *RWC* was determined as previously described [42]. First, the plants were weighed (M1) before treatment, then, weighed after different treatments (M2). Finally, after 30 min at 110°C, plants were dried overnight at 55°C until the mass (M3) no longer changed and the *RWC* (%) was calculated. *RWC* (%) = (M2 − M3)/(M1 − M3) × 100.

### Real-time polymerase chain reaction (qRT-PCR) analysis

The cabbage leaves of the six treatment groups in Table 1 were taken respectively, and the total RNA in the leaves was extracted with TRIzol (Invitrogen, California, USA), and reverse transcribed using the 5 × All-in-one MasterMix (ABM, Canada). Using cDNA as a template and cabbage *ACTIN* gene as an internal reference, a Bio-Rad CFX96 PCR detection system (Bio-Rad, California, USA) was used to detect the changes in the expression levels of BrRuBisCO enzyme-related genes.

### Biotin-switch assay (BSA) for determining S-sulfhydration

The purified protein was treated with H_2_S, and S-sulfhydration was detected by the biotin switch method, as described briefly [9]. Recombinant proteins were first purified by nickel affinity chromatography and then incubated with 2 mmol·L^−1^ NaHS or 2 mmol·L^−1^ Dithiothreitol (DTT) for 30 min at 4°C. Proteins were precipitated with pre-chilled acetone and dissolved in 100 μL HEN buffer. Next, 400 μL of methyl methanethiosulfonate blocking solution was added and the solution was incubated at 50°C for 25 min. The protein was then precipitated with pre-cooled acetone and the acetone precipitate dissolved in 100 μL HEN buffer with 1% SDS and 30 μL biotin- N-(6-(biotinamido)hexyl)-3′-(2′-pyridyldithio)-propionamide solution and incubated at 25°C for three hours. Finally, the samples were separated by SDS-PAGE electrophoresis and the proteins were transferred to a nitrocellulose membrane. Solutions were subjected to western blot analysis using biotin antibody, AP goat anti-mouse alkaline phosphatase label, and nitro blue tetrazolium/5-bromo-4-chloro-3-indolyl phosphate color detection. The data were analyzed using Image J software.

### BrRuBisCO enzyme carboxylation activity assay

The spectrophotometric enzyme-coupled assay was used as previously described with some adjustments [41, 43]. The total protein of the leaves under six different treatments was obtained and the changes of RuBisCO carboxylation activity in different treatments were measured after leveling the protein concentration, calculated as follows: RuBisCO carboxylation activity = [(*ΔA*_determination_ − *ΔA*_control_)*V*_reaction system_ × 1000]/(2 × 6.22 × *Δtm*).

Note: *ΔA*_determination_ measures the change in OD_340_ of the sample for 1 min, *ΔA*_control_ measures the change in OD_340_ of the control for 1 min, *V*_reaction system_ measures the volume of the reaction system, 2 means two mol of NADH is oxidized for every one mol of CO_2_ immobilized in RuBisCO, 6.22 is the extinction coefficient of 1 μmol·L^−1^ NADH at 340 nm, *Δt* is the interval time corresponding to *ΔA and* m is the addition of 10 μg protein per reaction.

### Determination of stomatal apertures

The stomatal aperture measurement method has been described previously [25]. The stomatal opening was measured by taking the lower epidermis of one-month-old normal-growing cabbage leaves and incubated with epidermal strip buffer containing 10 mmol·L^−1^ MES and 50 mmol·L^−1^ KCl and kept at 23°C under light for three hours for later use. The reserve epidermal strips were treated in the following Petri dishes for 15 min (Table 2), and all reagents were prepared with MES buffer. After treatment, the stomatal status of epidermal strip was observed under the light microscope. The computer software was used to take stomatal pictures and measure stomatal aperture. Data between different treatments were measured from at least 60 guard cells, and at least three independent replications were performed.

### Measurements of net cl^−^, K^+^, and H^+^ fluxes

Net fluxes of Cl^−^, K^+^, and H^+^ were determined in the YoungerUSA (Xuyue Beijing) NMT Service Center using Non-invasive Micro-test Technology and iFluxes/imFluxes 1.0 Software (NMT100 Series, YoungerUSA LLC, Amherst, MA, USA; Xuyue (Beijing) Sci. & Tech. Co., Ltd, Beijing, China) as described [25, 44]. One-month-old normal-growing leaf epidermis of cabbage was taken, plated and soaked in buffer containing strips of the epidermis with 10 mmol·L^−1^ MES and 50 mmol·L^−1^ KCl under light for three hours before use. Then the epidermal strip was fixed to the bottom of a disposable 5 mL Petri dish for the test, and 3 mL of the treatment reagent was added, as shown in Table 2 (the difference was that all treatment reagents were prepared in the test buffer provided by the tester, for example, the reagent added in the CK group was 3 mL of test buffer), and the processing time was 15 min. Each treatment for each ion contained three replicates. After sample processing, rinse 3–5 times with test buffer, fill up the test buffer and balance for 15 min before going on the machine. Read the electrode data of guard cell membrane randomly selected on the micro-operating table for at least 6 min and every 6 s. The test solution of Cl^−^ was composed of the following: 0.05 mmol·L^−1^ KCl, 0.05 mmol·L^−1^ CaCl_2_, 0.05 mmol·L^−1^ MgCl_2_, 0.25 mmol·L^−1^ NaCl and 0.2 mmol·L^−1^ Na_2_SO_4_ at pH 6.0. The test solution for K^+^ and H^+^ was composed of the following: 0.1 mmol·L^−1^ KCl, 0.1 mmol·L^−1^ CaCl_2_, 0.1 mmol·L^−1^ MgCl_2_, 0.5 mmol·L^−1^ NaCl, 0.3 mmol·L^−1^ MES and 0.2 mmol·L^−1^ Na_2_SO_4_ at pH 6.0. Electrode filling solution with three ions (K^+^ and Cl^−^: 100 mmol·L^−1^ KCl; H^+^: 40 mmol·L^−1^ KH_2_PO_4_, 15 mmol·L^−1^ NaCl, pH 7.0). Finally, the net ion flow was statistically analysed, with positive values representing outflow and negative values representing inflow.

### Statistical analyses

Three technical replicates were performed for each sample, and the mean ± SE represents the actual data. The software used for one-way ANOVA analysis of the data was SPSS (version 17, IBM SPSS, Chicago, IL, USA). After Tukey’s test (*P* < 0.05), the results with significant differences were shown in different black lowercase letters.

## Supplementary Material

Web_Material_uhac284

## Data Availability

All relevant data can be found in the manuscript and supplementary materials.
