# Supplementary material for: H2S-mediated balance regulation of stomatal and non-stomatal factors responding to drought stress in Chinese cabbage
Source: Hortic Res. 2022 Dec 23;10(3):uhac284. doi: 10.1093/hr/uhac284 (PMC10018781; doi:10.1093/hr/uhac284)
Supplement: Web_Material_uhac284 [file web_material_uhac284.zip › Supplementary Figures 12.8.pptx]

## Slide 1
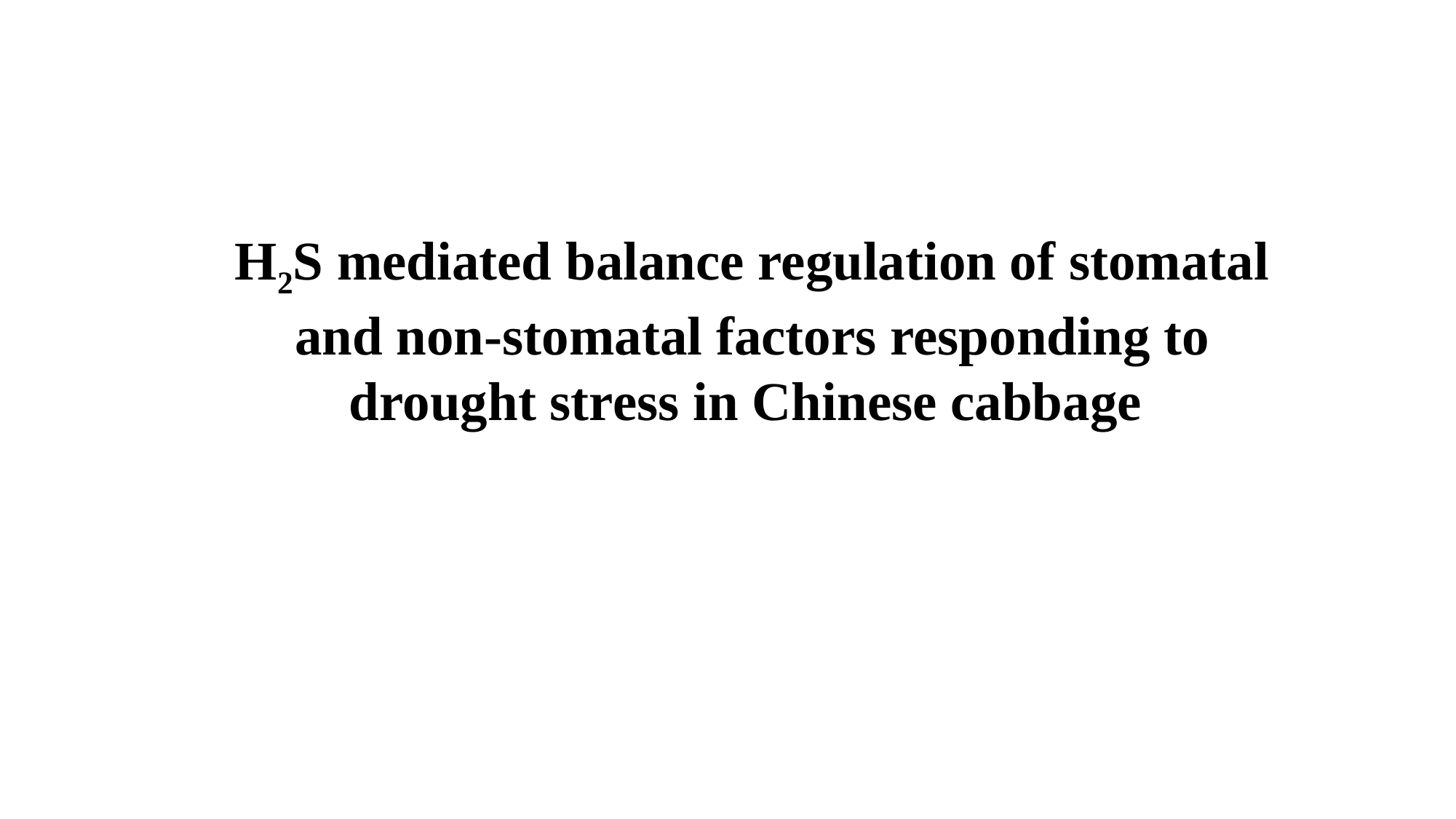

H2S mediated balance regulation of stomatal and non-stomatal factors responding to drought stress in Chinese cabbage

## Slide 2
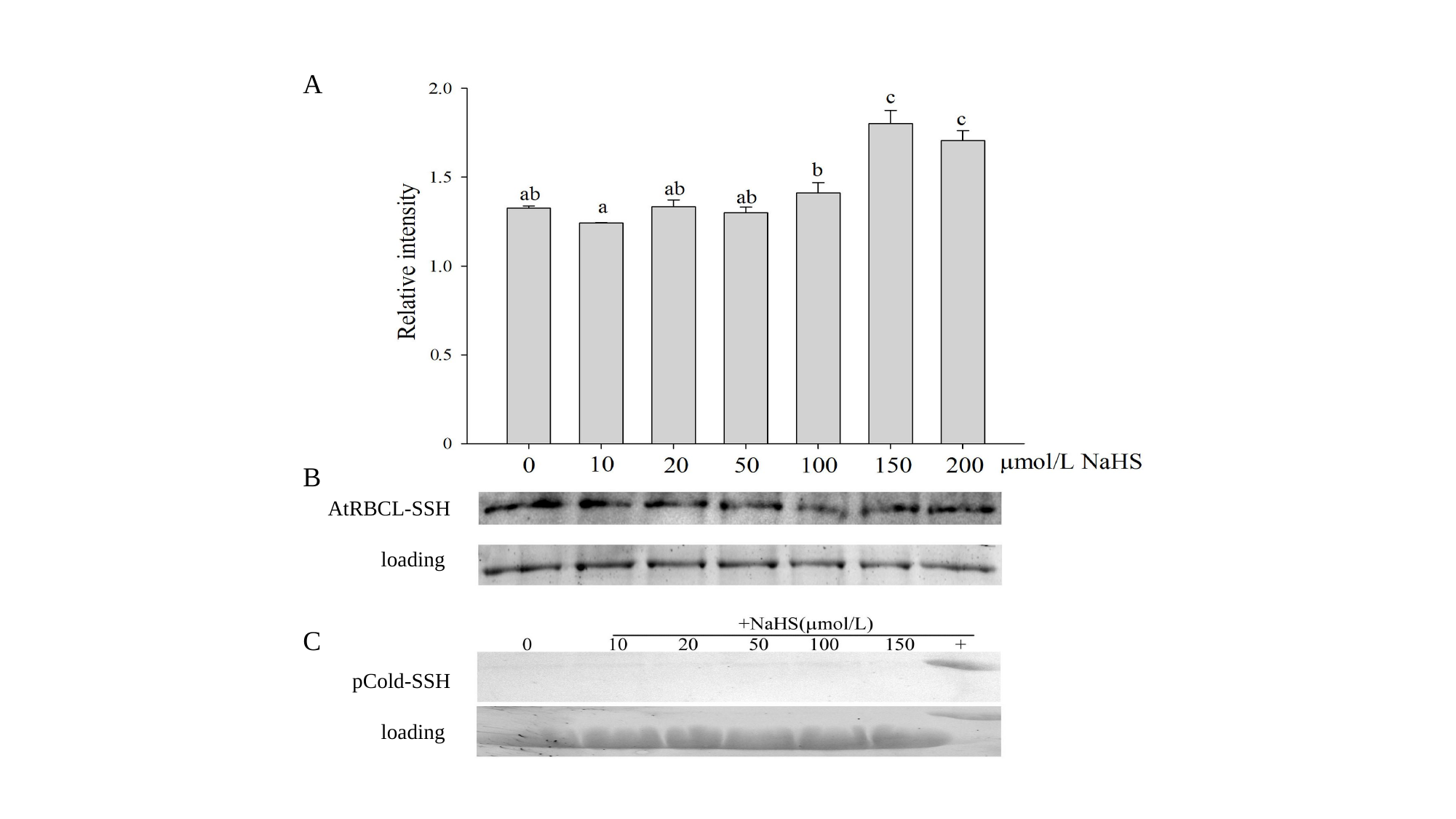

AtRBCL-SSH
loading
pCold-SSH
loading
A
B
C

## Slide 3
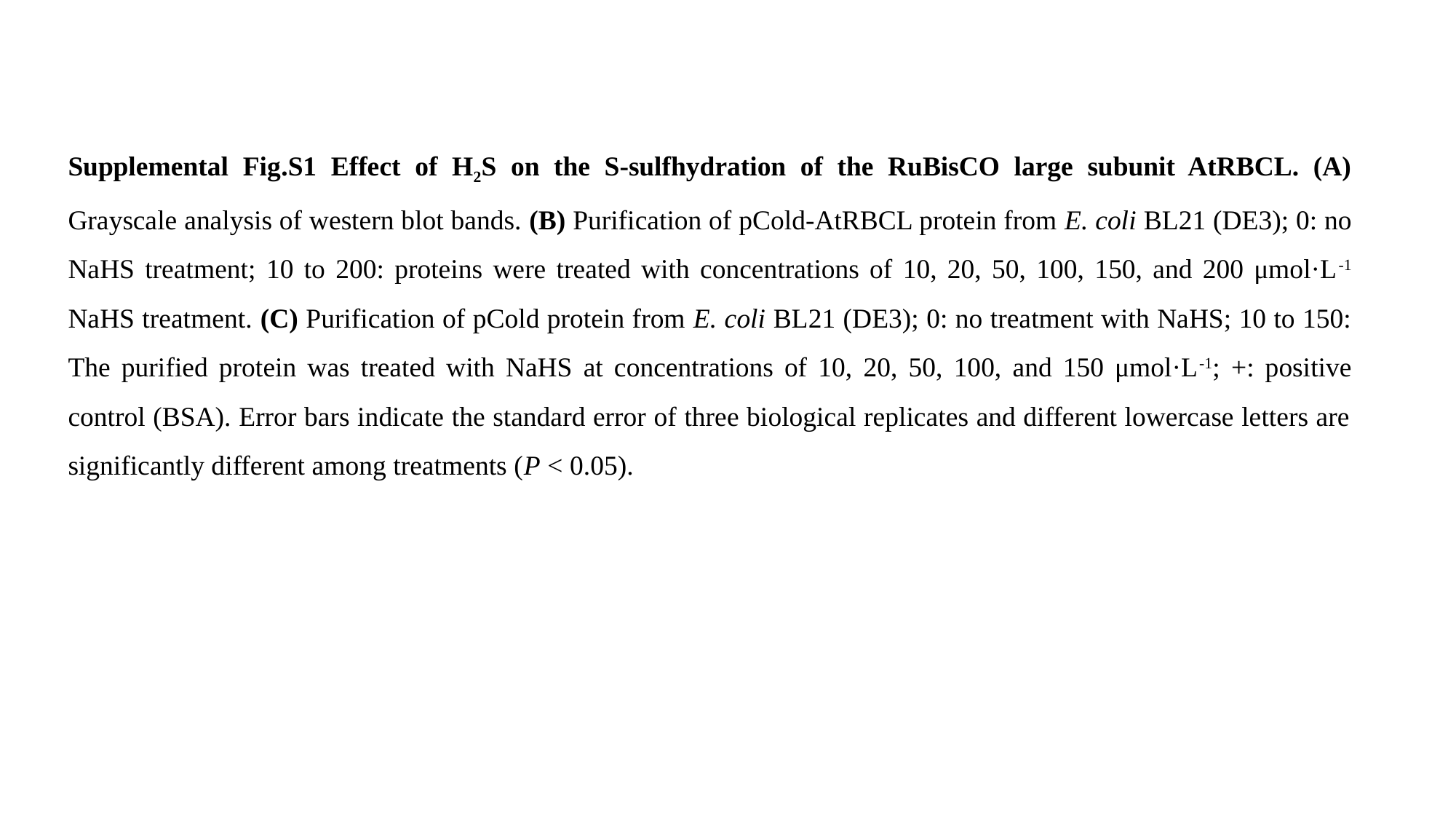

Supplemental Fig.S1 Effect of H2S on the S-sulfhydration of the RuBisCO large subunit AtRBCL. (A) Grayscale analysis of western blot bands. (B) Purification of pCold-AtRBCL protein from E. coli BL21 (DE3); 0: no NaHS treatment; 10 to 200: proteins were treated with concentrations of 10, 20, 50, 100, 150, and 200 μmol·L-1 NaHS treatment. (C) Purification of pCold protein from E. coli BL21 (DE3); 0: no treatment with NaHS; 10 to 150: The purified protein was treated with NaHS at concentrations of 10, 20, 50, 100, and 150 μmol·L-1; +: positive control (BSA). Error bars indicate the standard error of three biological replicates and different lowercase letters are significantly different among treatments (P < 0.05).

## Slide 4
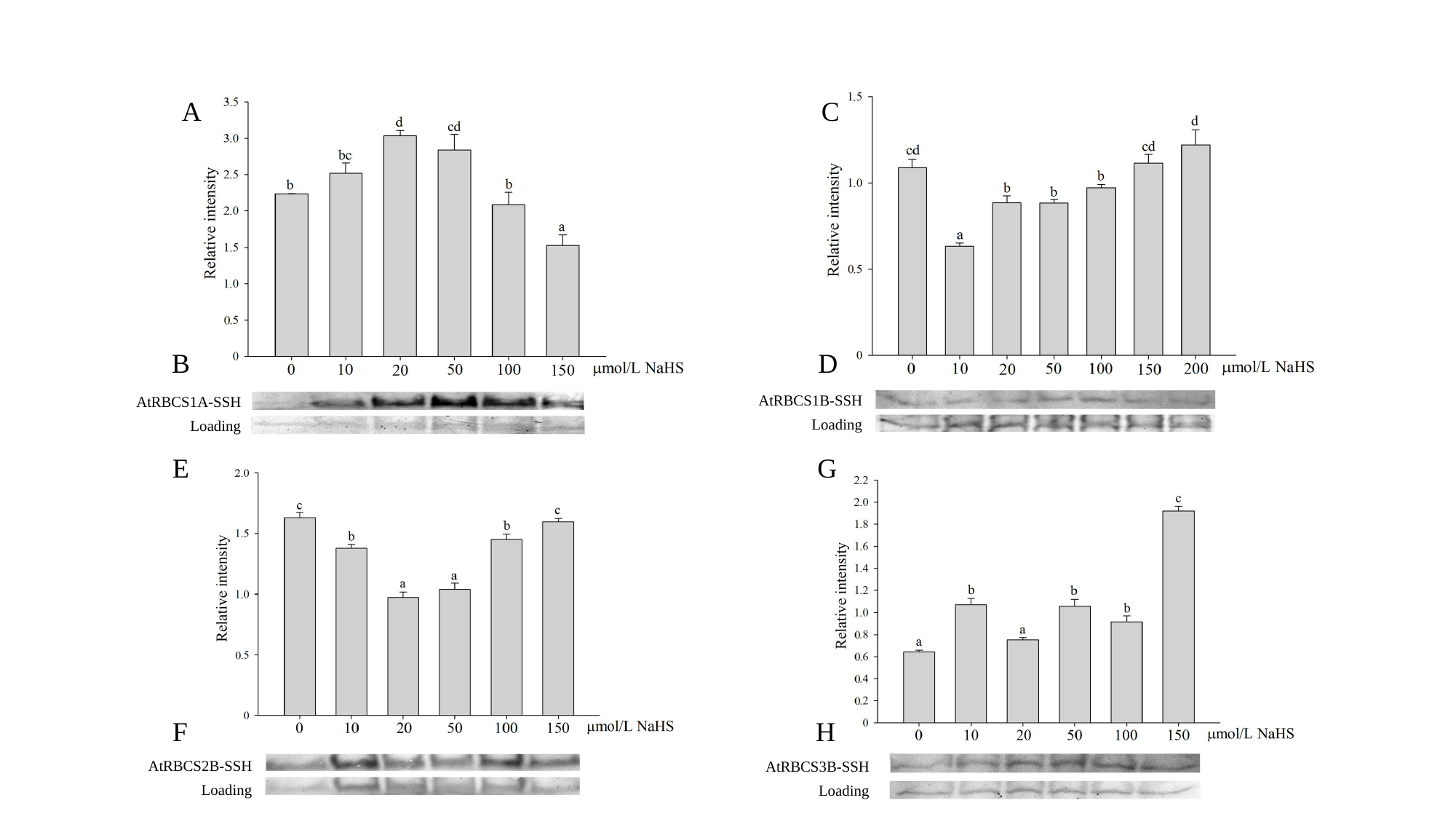

AtRBCS1B-SSH
Loading
AtRBCS1A-SSH
Loading
AtRBCS2B-SSH
Loading
AtRBCS3B-SSH
Loading
 A C
 E G
 B D
 F H

## Slide 5
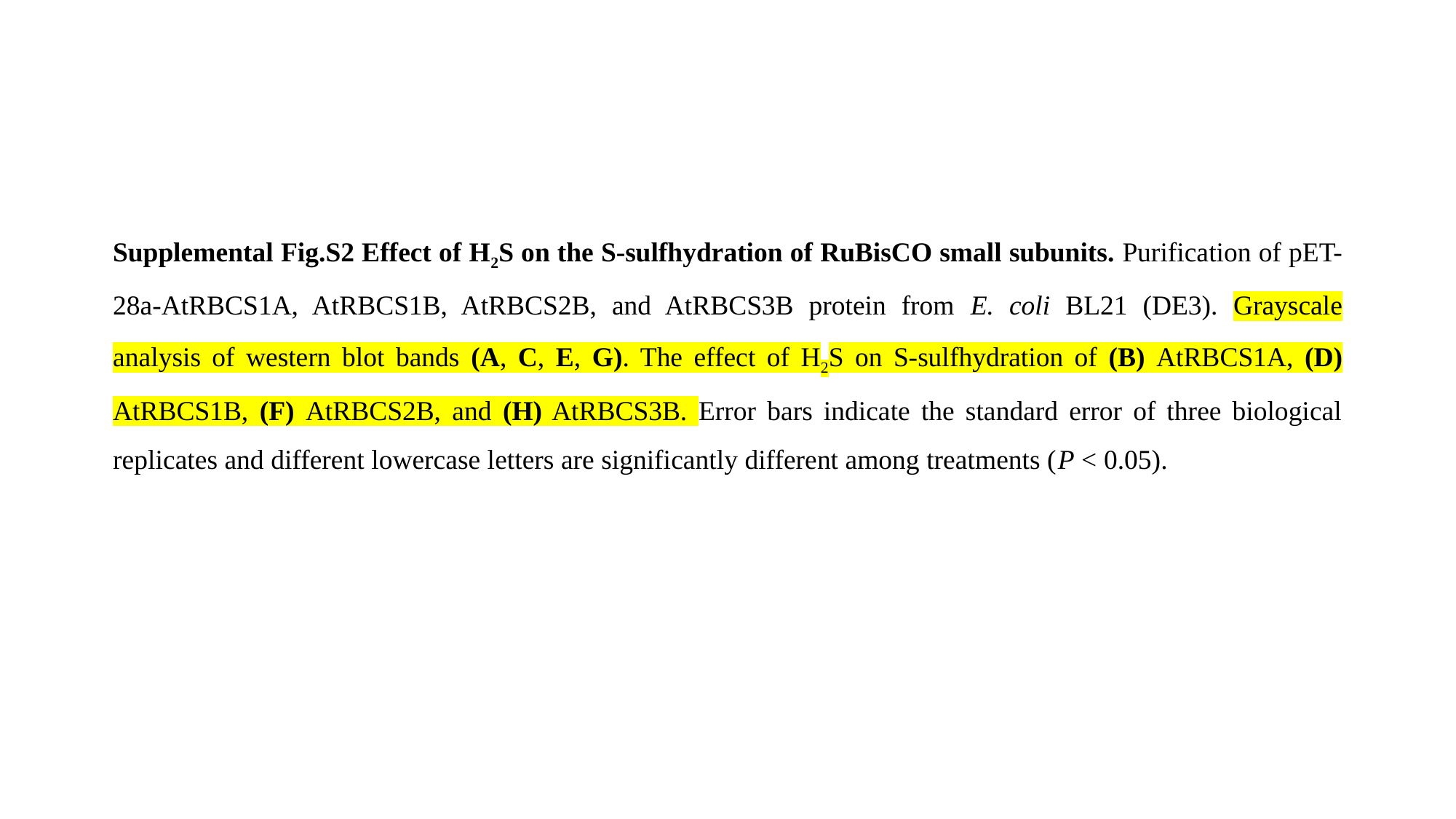

Supplemental Fig.S2 Effect of H2S on the S-sulfhydration of RuBisCO small subunits. Purification of pET-28a-AtRBCS1A, AtRBCS1B, AtRBCS2B, and AtRBCS3B protein from E. coli BL21 (DE3). Grayscale analysis of western blot bands (A, C, E, G). The effect of H2S on S-sulfhydration of (B) AtRBCS1A, (D) AtRBCS1B, (F) AtRBCS2B, and (H) AtRBCS3B. Error bars indicate the standard error of three biological replicates and different lowercase letters are significantly different among treatments (P < 0.05).

## Slide 6
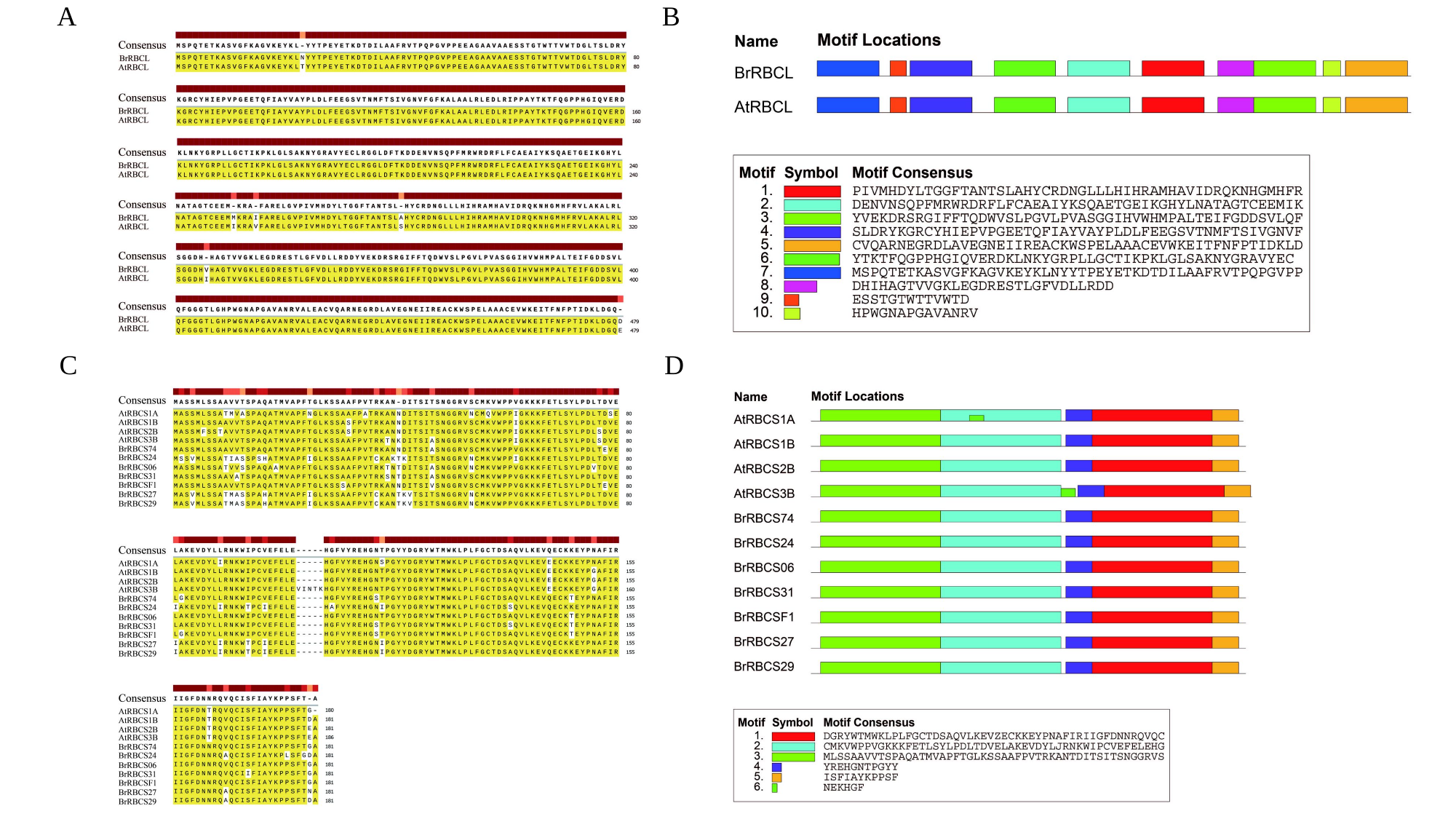

A B
C D

## Slide 7
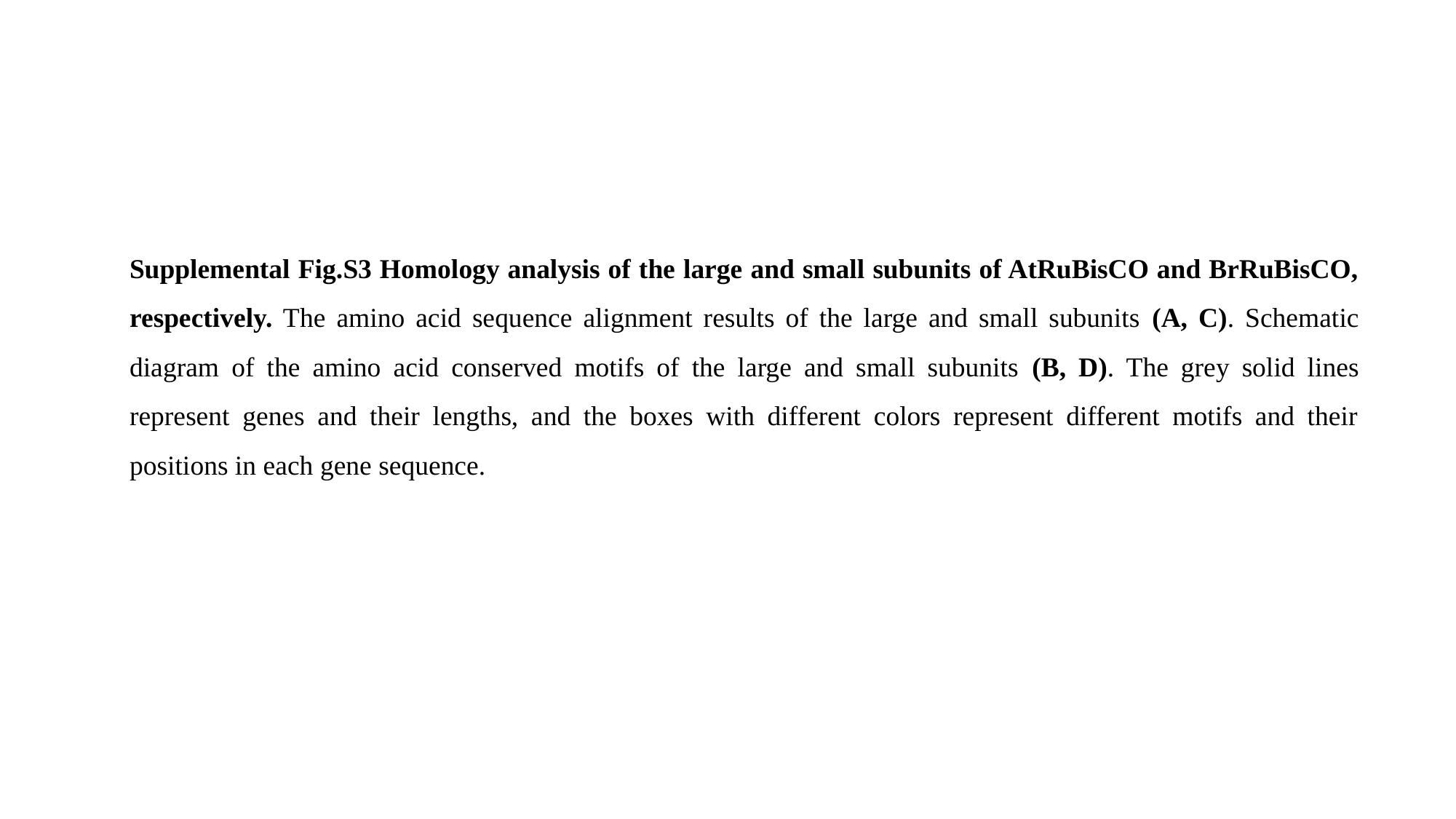

Supplemental Fig.S3 Homology analysis of the large and small subunits of AtRuBisCO and BrRuBisCO, respectively. The amino acid sequence alignment results of the large and small subunits (A, C). Schematic diagram of the amino acid conserved motifs of the large and small subunits (B, D). The grey solid lines represent genes and their lengths, and the boxes with different colors represent different motifs and their positions in each gene sequence.

## Slide 8
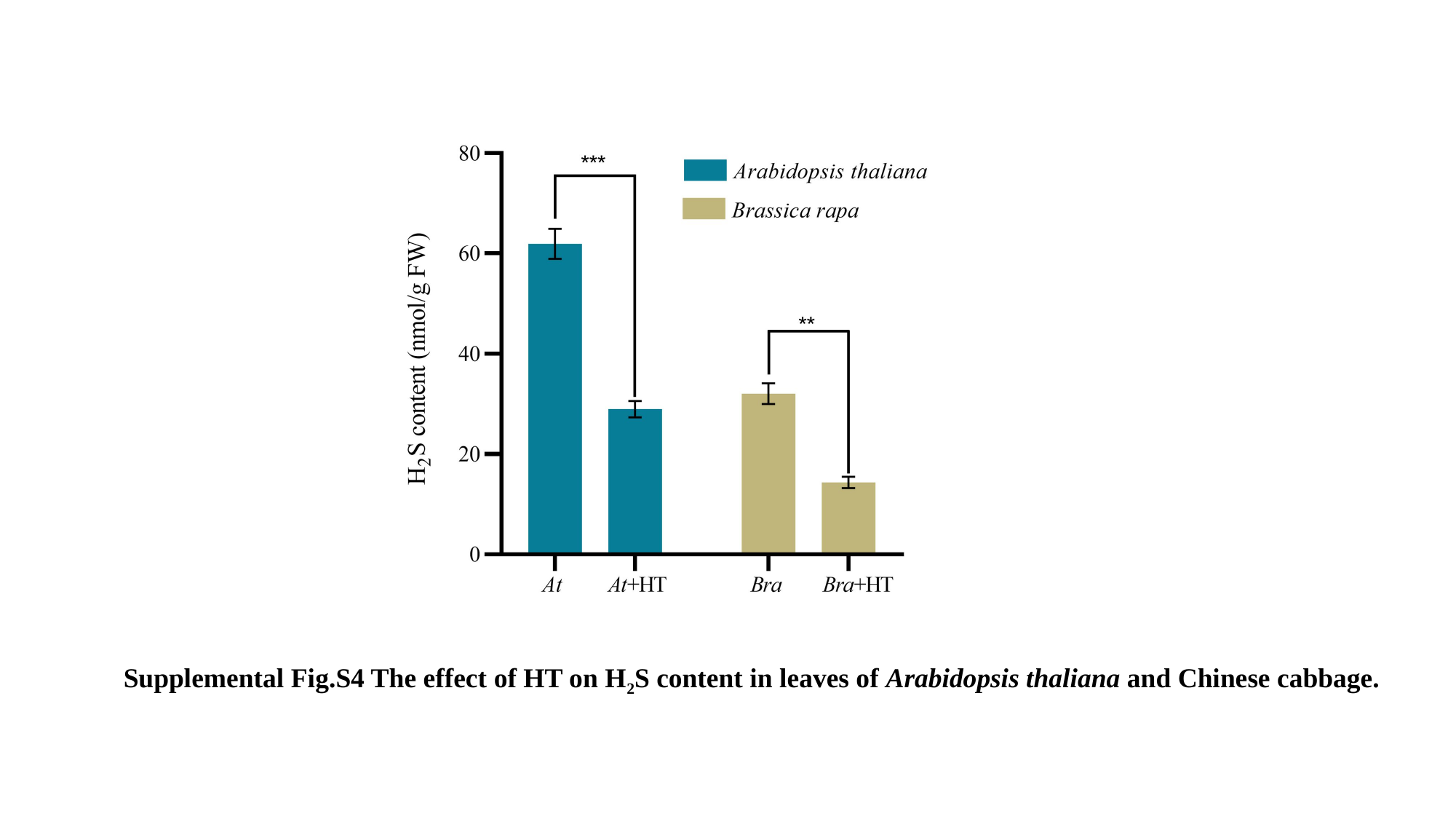

Supplemental Fig.S4 The effect of HT on H2S content in leaves of Arabidopsis thaliana and Chinese cabbage.
